# PAFR selectively mediates radioresistance and irradiation-induced autophagy suppression in prostate cancer cells

**DOI:** 10.18632/oncotarget.14647

**Published:** 2017-01-14

**Authors:** Bing Yao, Bingqian Liu, Lei Shi, Xiang Li, Chuanchuan Ren, Mingbo Cai, Wen Wang, Jianhua Li, Yongde Sun, Yudong Wu, Jianguo Wen

**Affiliations:** ^1^ Department of Urology, The First Affiliated Hospital of Zhengzhou University, Zhengzhou, China; ^2^ Department of Plastic Surgery, The First Affiliated Hospital of Zhengzhou University, Zhengzhou, China; ^3^ Department of Gynaecology and Obstetrics, The First Affiliated Hospital of Zhengzhou University, Zhengzhou, China

**Keywords:** platelet activating factor receptor, prostatic neoplasms, radiotherapy, autophagy, drug sensitivity

## Abstract

Platelet-activating factor receptor (PAFR) promotes tumorigenesis, angiogenesis and metastasis. Here, we defined the PAFR as a yielding new inhibiting target to selectively enhance the sensitivity of prostate cancer (PCa) cells to radiation. The selective responding to PAFR inhibiter may be caused by the differential expression pattern of PAFR in PCa cells. In this study, we also determined PAFR as a molecular basis by which the radiation induces autophagy suppression independent of activating mTOR pathway. PAFR can bind to the autophagy-indispensable protein Beclin 1, leading to the disability in its serine phosphorylation. The PAFR antagonist Ginkgolide B (GB) can sensitize radiotherapy by disrupting the formation of PAFR/Beclin 1 complex in PC3 and LNCaP cells, which have elevated PAFR expression after radiation exposure. Most importantly, GB efficiently radiosensitized PC3 and LNCaP tumor xenografts *in vivo*, and significantly reduced tumor burden. Overall, our results elucidated a significant role of GB in selectively improving the outcomes of PCa receiving radiation therapy.

## INTRODUCTION

Prostate cancer (PCa) is the second leading cause of cancer deaths and the most common cancer in men in the United States [1], and its morbidity is also rapidly rising in East Asian [2]. During the PSA era, most PCa can be diagnosed at an early stage, for which radiotherapy is one of the standard treatment modalities, alone or combined with androgen deprivation therapy (ADT) [3, 4]. Because of the side-effect of radiation, limited dose of less than 85 Gy can be delivered to prostate. However, approximately 10% to 45% PCa are radioresistant either not responding or relapsing after receiving limited dose irradiation [5–7]. This supports that an alternative to improving efficacy of radiation would be to use radiation-sensitizer not to increase radiation dose.

G-protein-coupled receptor (GPCR) is a large family of cell-surface proteins [8, 9]. The abnormal expression of GPCR or aberrant activation by their ligands would initiate their signaling networks and PCa progression and resistance to anti-cancer treatments. Platelet-activating factor (PAR) receptor (PAFR) is one of GPCR, which can be activated by PAR. It has been reported to promote non-small cell lung cancer (NSCLC) progression and metastasis by initiating forward feedback loop between PAFR and STAT3 [10], and contributes to the malignant development of esophageal squamous cell carcinoma by stimulating PI3K/AKT activation [11]. PAFR can also induce chemotherapy resistance in ovarian cancer through transactivating of epidermal growth factor receptor (EGFR) [12, 13]. In addition, LNCaP and PC-3 cells have been shown to produce PAF endogenously [14]. However, the biologic roles of PAFR in PCa progression and radioresistance have not been investigated.

## RESULTS

### PAFR inhibition sensitizes PC3 and LNCaP cells to irradiation

**Figure 1:**
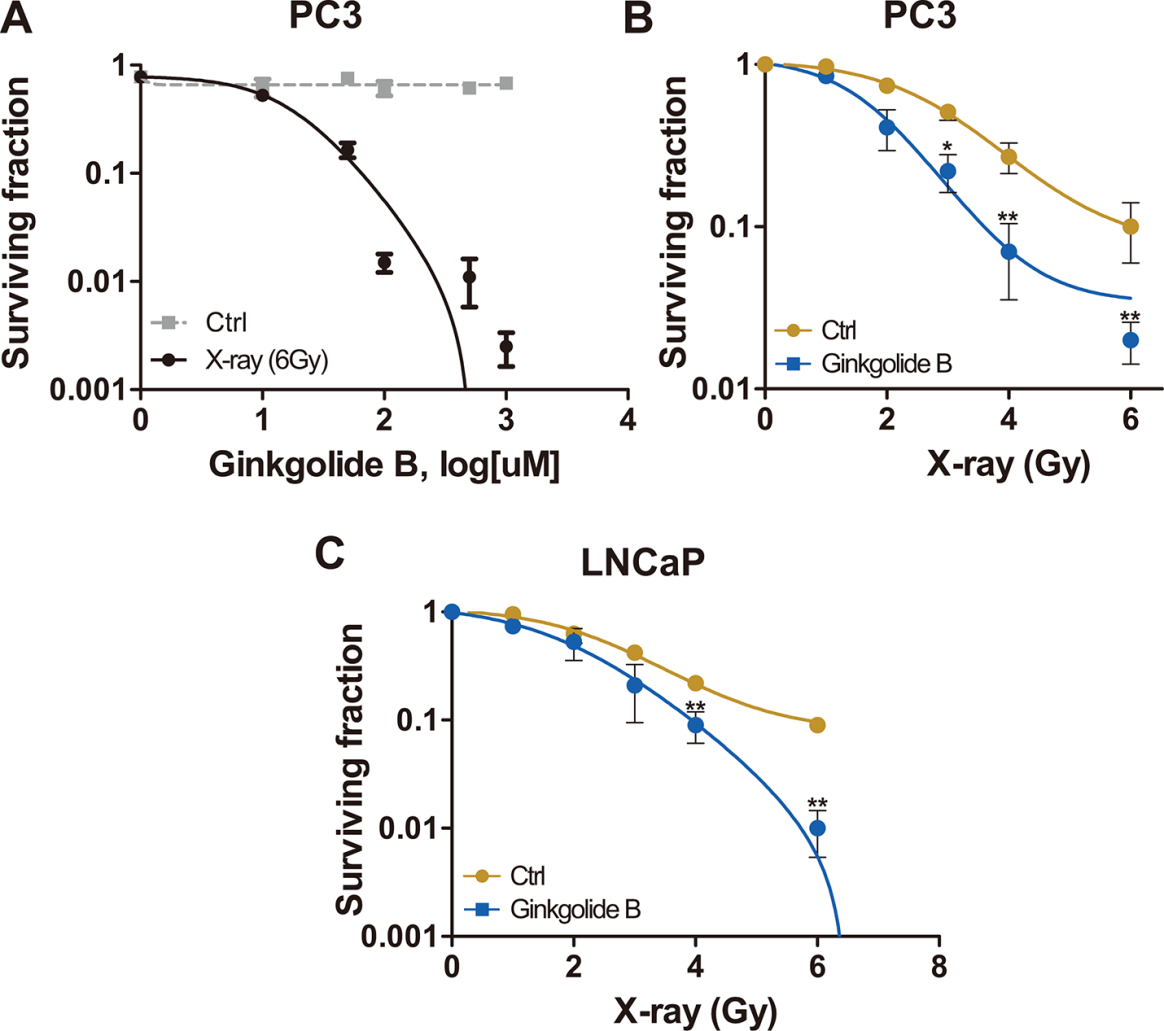
PAFR inhibition sensitizes PC3 and LNCaP cells to irradiation (**A**) Cells received treatment with ladder concentrations of Ginkgolide B (GB, 1–1000 μM) following control (Ctrl) or irradiation with 6 Gy X-rays and subjected to the clonogenic survival assay. Surviving fractions were calculated with non-line dose-response profiles and normalized by the effects of drug treatment alone. (**B**) Surviving fractions of PC3 cells received treatment of GB and irradiation. Cells were irradiated and treated with 100 μM GB or DMSO (Ctrl), and delivered for clonogenic survival assay. Surviving fractions were calculated with non-line dose-response profiles and normalized by the effects of drug treatment alone. Data represents at least 3 independent experiments. *P < 0.05, **P < 0.01. (**C**) Surviving fractions of LNCaP cells received the treated of GB and irradiation. Cells were irradiated and treated with 100 μM GB or DMSO (Ctrl), and delivered for clonogenic survival assay.

To test whether inhibition of PAFR enhance the sensitivity of PCa to ionizing irradiation, we chose a castration-resistant and highly metastatic (PC3) and a androgen-dependent and lowly metastatic prostate adenocarcinoma cell lines. We measured cell-killing effects of ionizing radiation in PCa cells by examining colony-forming ability (Clonogenic survival assay). PC3 cells were treated with a series of concentrations of PAFR antagonist, Ginkgolide B (GB), post sham (Ctrl) and genuine irradiation. As shown in Figure 1A, it seems that GB did not show any suppressive activity against PC3 cells. However, it sensitized PC3 cells to irradiation (6 Gy) in manner of dose dependent, approximately reaching a maximum at 100μM of treatment. At this concentration, GB significantly enhanced cell-killing effects of radiation in both PC3 and LNCaP cells, resulting in significant decrease in surviving fraction at 6 Gy of X-ray (Figure 1B and 1C).

### Ginkgolide B enhances the effects of irradiation on inducing apoptosis and impeding proliferation in prostate cancer cells

**Figure 2:**
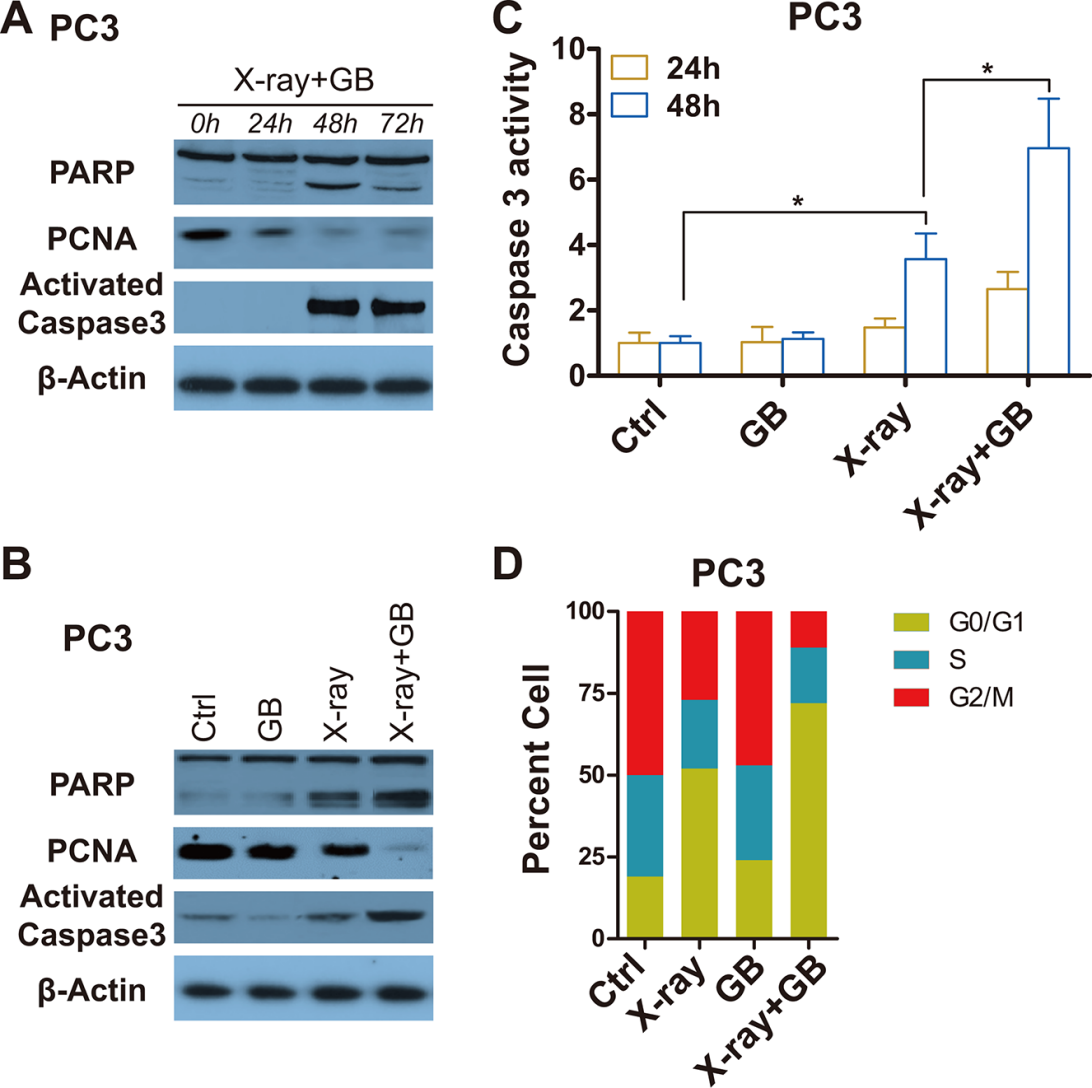
GB enhances the effects of irradiation on inducing apoptosis and impeding proliferation in prostate cancer cells (**A**) Representative western blot analysis of cleaved PARP, PCNA, activated caspase 3 and β-actin in PC3 cells received irradiation (6 Gy) followed by treatment with 100 μM GB for indicated times (post-irradiation). (**B**) Representative western blot analysis of cleaved PARP, PCNA, activated caspase 3 and β-actin in PC3 cells treated by 100 μM GB for 48 hours post-irradiation. (**C**) Caspase 3 activity in PC3 cells treated by 100 μM GB for 24 hours or 48 hours post-irradiation. Signals were normalized to the fluorescence of sham-treated controls (Ctrl). Data represents at least 3 independent experiments. *P < 0.05. (**D**) Cell cycle distributions in PC3 cells treated by 100 μM GB for 48 hours post-irradiation. Data represents at least 3 independent experiments.

After radiation exposure, treatment of PC3 cells with GB for 48 hours (h) resulted in significantly mroe apoptosis and less proliferation, shown by the increased apoptotic markers, cleaved poly adp-ribose-polymerase (cPARP) and activated caspase 3, and decreased proliferative marker, proliferating cell nuclear antigen (PCNA) (Figure 2A). As shown in Figure 2B, combination therapy of radiation with GB induced more apoptosis and weakened proliferation compared to radiation monotherapy (Figure 2B). Consistent with these observations, there was a statistically significant increase in caspase 3 activity in cells treated with X-ray (*P* < 0.05), and the most increase was observed in the groups received combination therapy when compared with sham and GB treatment (*P* < 0.05, Figure 2C). In addition, cell cycle assay was conducted by flow cytometry, results showed that GB reduced cells in G2/M and S stages (Figure 2D). It is worth to note that treatment of GB alone in the culture medium failed to induce cellular apoptotic death (Figure 2B–2D).

### Ginkgolide B fails to sensitize prostate cancer cells to irradiation in the absence of PAFR

**Figure 3:**
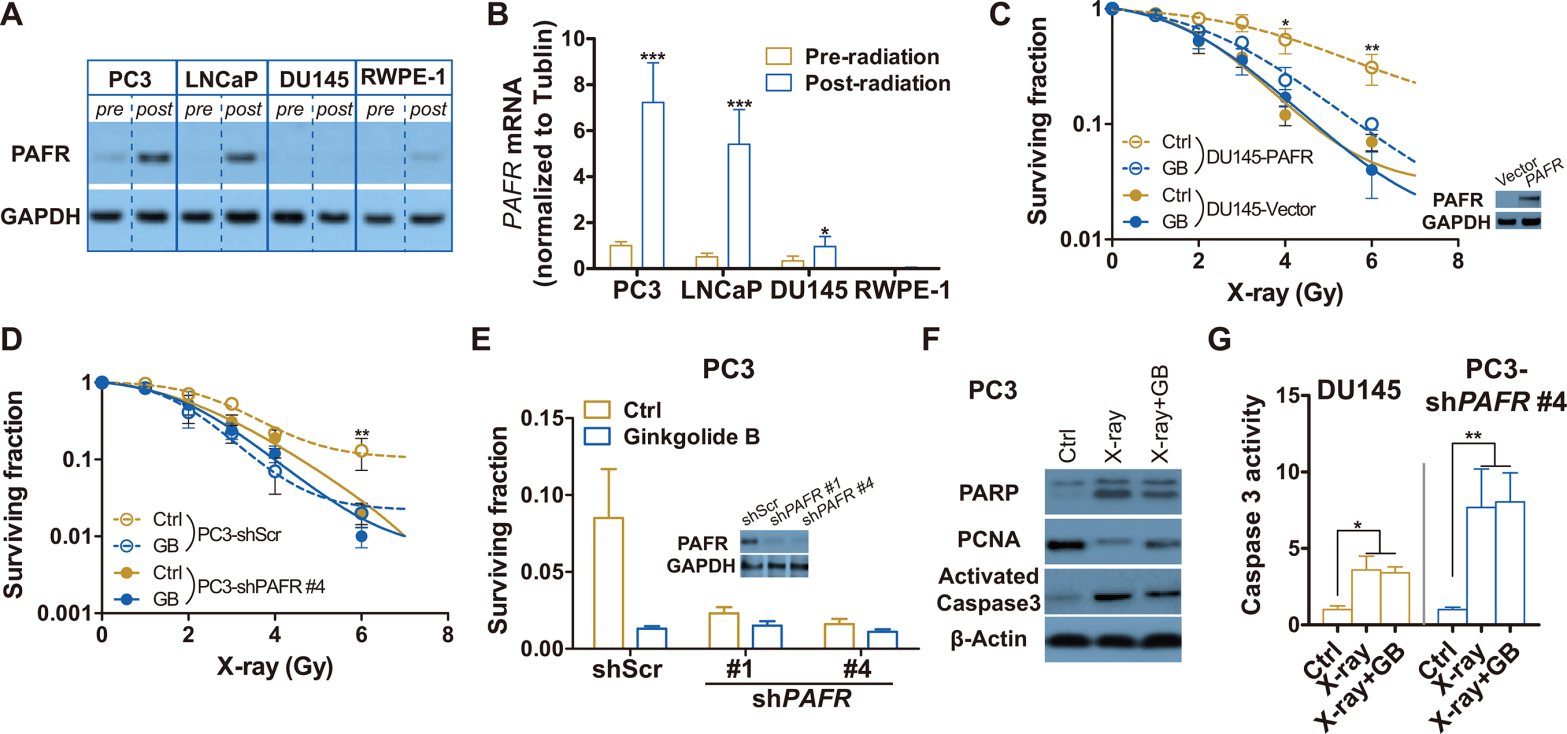
PAFR inhibition fails to sensitize DU-145 and PAFR-knockdowned PC3 (PC3-shPAFR) cells to irradiation (**A**) Representative western blot analysis of the expressions of PAFR protein in PC3, LNCaP, DU-145 and RWPE-1 cell lines pre- and 24 h post-irradiation (6 Gy X-rays). (**B**) RT-PCR analysis of the expressions of PAFR mRNA in PC3, LNCaP, DU-145 and RWPE-1 cell lines pre- and 24 h post-irradiation (6 Gy X-rays). (**C**) Surviving fractions of DU145-vector or DU145-PAFR cells received treatment of GB and irradiation. Cells were irradiated and treated with 100 μM GB or DMSO (Ctrl), and delivered for clonogenic survival assay. Surviving fractions were calculated with non-line dose-response profiles and normalized by the effects of drug treatment alone. Data represents at least 3 independent experiments. (**D**) Surviving fractions of PC3-shScr or PC3-shPAFR cells received the treated of GB and irradiation. Cells were irradiated and treated with 100 μM GB or DMSO (Ctrl), and delivered for clonogenic survival assay. (**E**) Surviving fractions of PC3-shPAFR cells received treatment of GB and irradiation. Cells were irradiated (6 Gy) and treated with 100 μM GB or DMSO (Ctrl), and delivered for clonogenic survival assay. The minimal graph is western blot analysis of the transfection effect of PAFR shock RNAs (shPAFRs). (**F**) Representative western blot analysis of cleaved PARP, PCNA, activated caspase 3 and β-actin in PC3-shPAFR cells treated by 100 μM GB for 48 hours post-irradiation. (**G**) Caspase 3 activity in DU-145 and PC3-shPAFR cells treated by 100 μM GB for 48 hours post-irradiation. Signals were normalized to the fluorescence of sham-treated controls (Ctrl). Data represents at least 3 independent experiments. *P < 0.05, **P < 0.01.

To additionally confirm that the GB-induced radiosensitization is specifically through PAFR inhibition, PAFR expressions before and after 6 Gy of X-ray are detected by western blot and RT-PCR analyses. Here, we confirm that PAFR is almost not expressed in unirradiated prostate cells and differentially expressed in irradiated prostate cells, showing that PAFR significantly overexpressed in X-ray exposed PC3 and LNCaP cells, but not in irradiated DU-145 and RWPE-1 (a non-oncogenic prostate epithelial cell line) (Figure 3A). mRNA levels of PAFR correlated with its protein levels (Figure 3B). As expected, GB fails to induce radiosensitization in DU145 cells because of little expression of PAFR after irradiation (Figure 3C). Stable PAFR overexpression makes DU145 cells (DU145-PAFR) resistant to radiation, and the effect of overexpressed PAFR mostly offseted by GB. To additionally validate the effect of GB on radiosensitization are mediated by PAFR, we stably knockdown PAFR in PC3 cells. Results in Figure 3D and 3E show that GB no longer induce radiosensitization in PAFR-silenced PC3 (PC3-shPAFR) cells. Moreover, Figure 3F and 3G show that GB don't further increase the apoptosis and reduce the proliferation of DU-145 and PC3-shPAFR caused by X-ray. At the last, we overexpress PAFR in DU145 cells, Whereas, In my opinion, the authors should use DU145 cell line to overexpress PAFR and show that makes cells resistant to radiation.

### Ginkgolide B enhances the antitumor activity of irradiation against xenografts formed by PC3 cells

**Figure 4:**
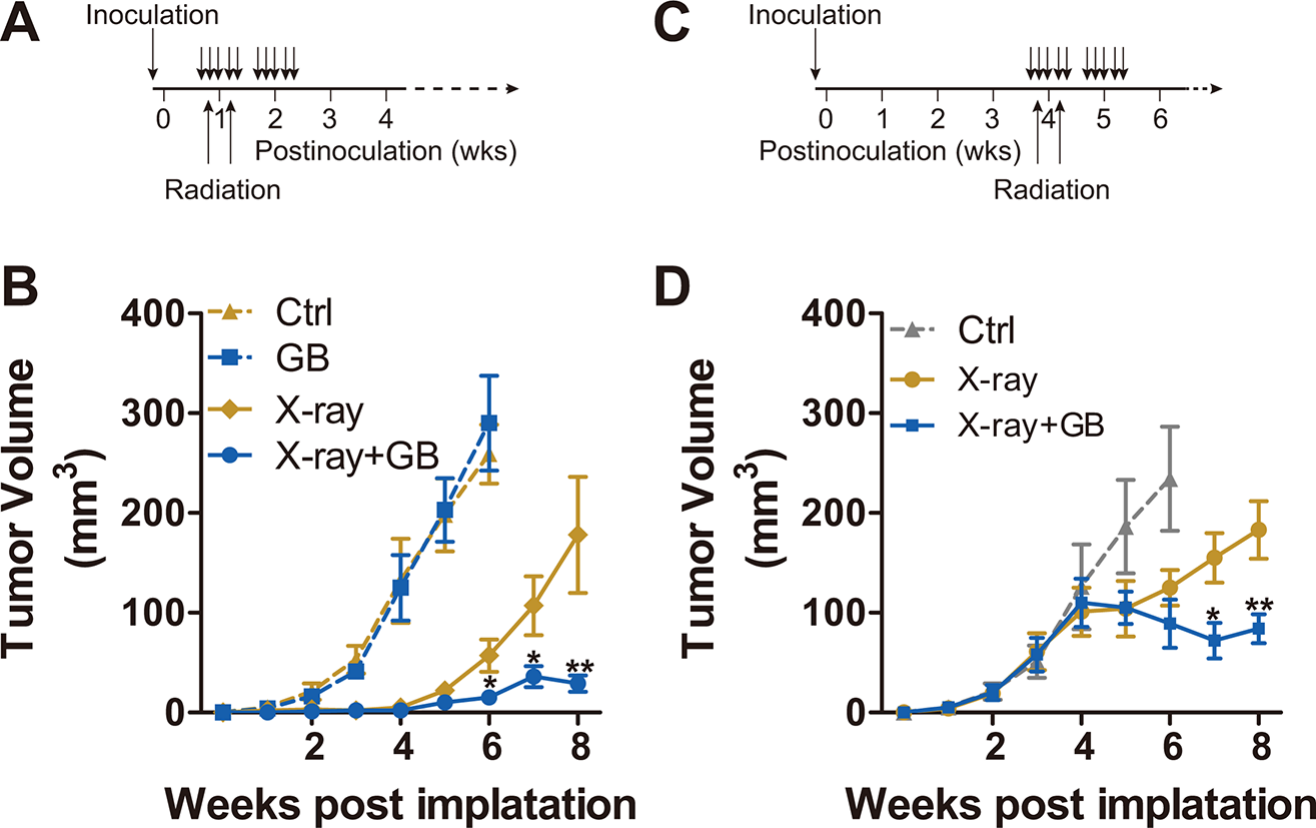
Ginkgolide B enhances the sensitivity of PC3 xenografts to irradiation (**A**) schematic description of the first *in vivo* experiment as detailed in the B. (**B**) The 107 PC3 cells per injection site were embedded in medium with 50% Matrigel, and injected subcutaneously into NOD-SCID mice. Five days postinoculation, mice were randomized, treated with GB (100 mg/kg) for 2 weeks (5 days per week), and irradiated (X-ray; 2.5 Gy delivered to xenografts area only) on second and fourth days of the first course of treatment. The monitoring on volume of the xenografts was conducted weekly. The mice were sacrificed and the tumors were harvested 6 or 8 weeks postinoculation. Results are mean volume ± SEM for 8 to 10 mice per group. *P < 0.05; **P < 0.01 compared with radiation alone (one-factor ANOVA). (**C**) schematic description of the first *in vivo* experiment as detailed in the D. (**D**) the size of tumors were also assessed weekly, and the mice were randomized into 3 treatment groups. Treatment was conducted during week 4 and 5 postinoculation, as described in C. Results are mean volume ± SEM for 8 to 10 mice per group. *P < 0.05; **P < 0.01 compared with radiation alone (one-factor ANOVA).

To test the hypothesis that GB can enhance the sensitivity of PCa to irradiation *in vivo*, the subcutaneous PC3 tumor models are used. The schemas outlining *in vivo* experiments are presented in Figure 4A and 4C, and results can be found in Figure 4B and 4D. In the first experiment, mice are randomized 5 days postinoculation and the drug treatment and/or irradiation are started immediately thereafter (Figure 4A). For the second experiment, mice are randomized 4 weeks postinoculation and the drug treatment and/or irradiation are the same as the first experiment (Figure 4C). Both Figure 4B and Figure 4D show that GB alone has no significant effect on suppression of PC3 xenografts. Irradiation alone is partially efficacious, whereas the combined treatment (X-ray plus GB) shows the most protuberant effect, significantly reducing tumor growth (vs. X-ray alone, *P* < 0.01).

### Irradiation-induced increasement in PAFR inhibits Beclin 1 phosphorylation and autophagy

**Figure 5:**
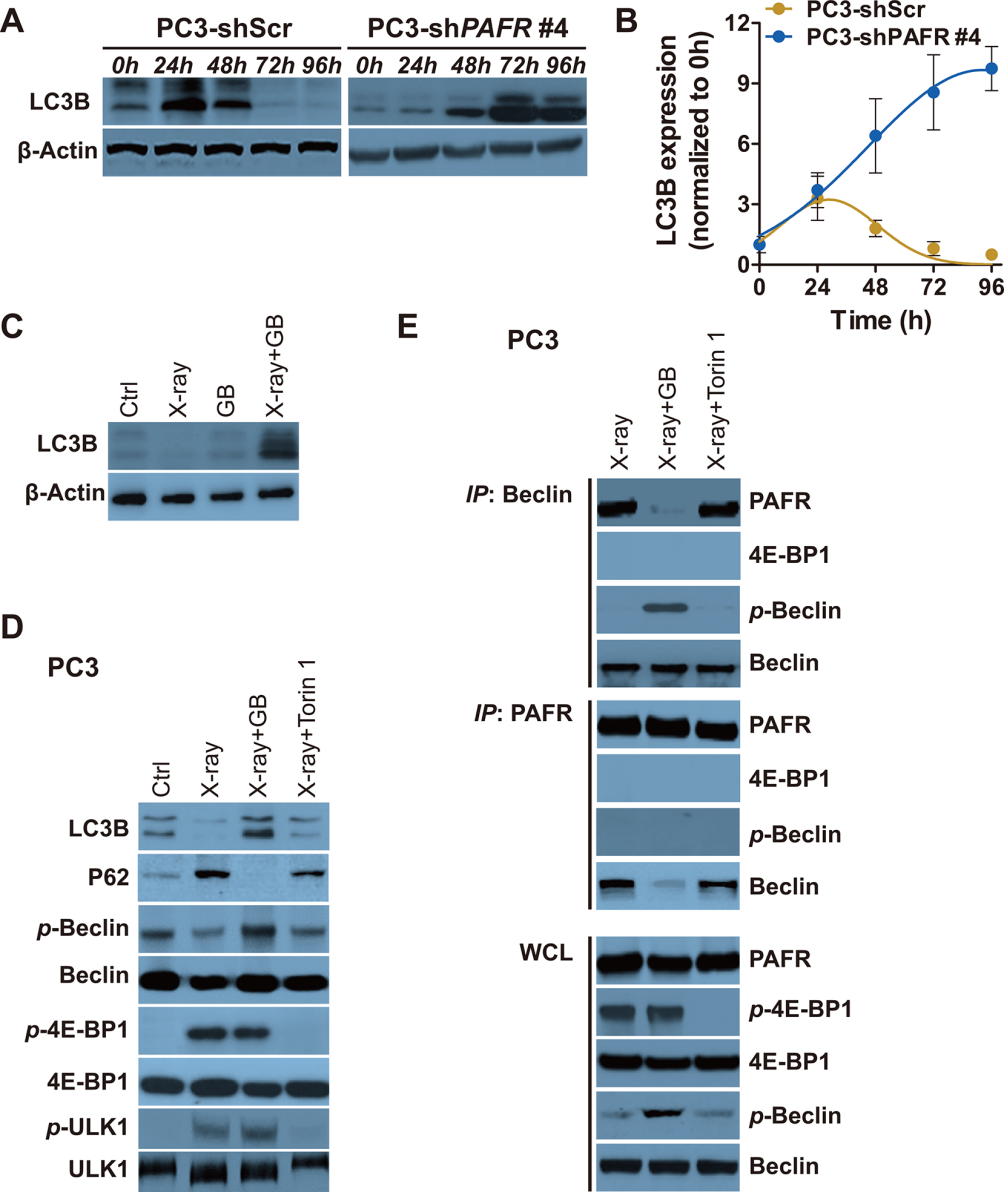
Irradiation-induced increasement of PAFR inhibits Beclin 1 phosphorylation and autophagy (**A**) Western blot analysis of LC3B in PC3 and PC3-shPAFR cells in indicated time postradiation. (**B**) Quantification of LC3B expression in PC3 and PC3-shPAFR cells in indicated time postradiation. The results are normalized by β-actin. Data represents at least 3 independent experiments. (**C**) Western blot analysis of LC3B in xenograft tumors harvested from Figure 4B. (**D**) Western blot analysis of LC3B, P62, p-Beclin 1, total Beclin 1, p-4E-BP1, total 4E-BP1 (21KD-band), p-ULK1 and total ULK1 in DMSO or GB-treated treated PC3 72 hours (72 h) post sham or 6 Gy of X-ray. (**E**) Immunoprecipitation of Beclin 1 with PAFR (and the reverse direction CO-IP) in PC3 cells in conditions shown in (D).

To explore the potential mechanism of PAFR-induced radioresistance in PCa cells, we test autophagosome formation in a continuous time in scramble or PAFR shRNA stably transfected PC3 cells (PC3-shScr or PC3-shPAFR) after radiation exposure. Treatment of PC3-shPAFR cells with radiation results in a persistent increase in autophagy within 96 hours, whereas a transient rising is found in PC3-shScr cells, as shown by the LC3B protein accumulation (Figure 5A and 5B). To confirm the effects of radiation and GB on autophagosome formation *in vivo*, we used xenograft tumors from NOD-SCID mice mentioned in Figure 4B. All tumors were harvested at 6-week post implantation. X-ray exposure resulted in less autophagy induction, as evidenced by an decrease in LC3B expression (Figure 5C). GB combined with radiation significantly promoted autophagy induction, whereas GB alone failed to do (Figure 5C).

During serum starvation, autophagy can be induced by inhibiting the protein mammalian target of rapamycin (mTOR), a nutrient-responsive kinase [17–19]. To further evaluate whether the activation of mTOR pathway is involved in irradiation-induced autophagy suppression, we used ATP-competitive inhibitor of mTOR, Torin 1 [20]. PC3 cells receive either GB or Torin 1, which requires 72 hours of radiation treatment for PAFR and mTOR pathway activation. Serine phosphorylation of Beclin 1 and the following autophagy is weakly increased by Torin 1 in X-ray treated PC3, as shown by accumulation of LC3B and reduction of P62 (Figure 5D). Beclin-1, mammalian homolog of yeast Atg6, is required for the autophagy induction. The effectiveness of Torin 1 is confirmed by suppressed phosphorylation of 4E-BP1 and ULK1, downstream proteins of mTOR kinase. However, a significant increase of autophagy is observed in cells received treatment of both radiation and GB (Figure 5D). In addition, GB results in the suppression the formation of PAFR/Beclin 1 complex (Figure 5E). More importantly, we find that PAFR can only bind to non-phosphorylated Beclin 1 (Figure 5E). These results remind us that increased PAFR may impede phosphorylation of Beclin 1 via directly binding to it.

## DISCUSSION

The high expression of PAFR and the corresponding ligand PAF results in the invasion and metastasis of colorectal cancer and NSCLC [10, 21]. Besides, several studies have shown that PAFR antagonists can reduce tumor metastasis *in vivo* [10, 21–23]. In addition, the upregulation of PAFR contributes to cisplatin resistance in ovarian cancer via activating PI3K and ERK pathways that lies downstream of activated PAFR [13]. Co-treatment with Ginkgolide B and cisplatin can markedly reduce tumor growth in an *in vivo* model of ovarian cancer [13]. However, it's unclear if PAFR leads to anticancer resistance in PCa. In this study, we demonstrate that PAFR inhibition can selectively enhances the sensitivity of radiotherapy in PCa cells with elevated PAFR expression after irradiation exposure. Mechanisms of PAFR-inhibition mediated sensitization to radiotherapy includes the weakness of proliferation, disruption of cell-cycle and the enhancement of radiation induced apoptosis. More importantly, our data clearly demonstrate that blocking PAFR using GB wouldn't disturb proliferation and cell-cycle movement in cancer cells growing in conditions without radiation exposure. This indicates the weak side-effect of PAFR inhibitor as an assistant medicine of radiotherapy.

Even though GB has few effect on cell growth of X-ray untreated PC3 and LNCaP cells, it can completely block radiation-induced P62 expression and remarkably reduce radiation-induced Beclin 1 serine phosphorylation and autophagosome formation (Figure 5D). The phosphorylated Beclin 1 plays an essential role in autophagy through forming complex with VPS34 and cross talking with various autophagy stimulatory or inhibitory molecules [24]. Although it has been known that activation of some cell surface GPCRs (e.g. T1R1/T1R3) suppresses autophagy via interfering mammalian target of rapamycin complex 1 (mTORC1) [25, 26], the expounded mechanism may not be applicable to all GPCRs. In this study, we demonstrate the elevated PAFR can bind to Beclin 1, impeding its serine phosphorylation and inactivation. Importantly, the effect only occurs in cells expressing PAFR after irradiation exposure.

Previous studies are conflicting in the effect of autophagy manipulation on radiosensitivity [27–29]. Causes for the conflicts may be including but not limited to (1) the different time of detecting autophagy post irradiation, (2) the use of cells with elevated PAFR expression after irradiation rather than that without alteration of PAFR expression in response to radiation, (3) the utilize of cell proliferation assays base upon mitochondrial activity rather than cell clonogenic survival assay base on directly detecting cell growth to determine the effect of autophagy induction on PCa cell survival. Moreover, a sequence of evidences support our results: (1) several lines proofs indicate that decreased Beclin 1 expression or activation results in tumor development [20, 30, 31]: Disable Mutant Beclin 1 can lead to autophagy suppression, anchorage-independent cell growth and enhance Akt-driven carcinogenesis. *In vivo*, allelic loss of Beclin 1 results in increment in the incidence of spontaneous malignancies in transgenic mice. In addition, xenograft NSCLCs formed by cells with mutated inactive form of Beclin 1 are resistant to both EGFR inhibitor and chemotherapy. (2) As a proverbial growth factor, epidermal growth factor (EGF) stimulates cell growth, proliferation, and differentiation by binding to its receptor EGFR [32]. Meanwhile, activated EGFR by EGF impedes the function of Beclin 1 and inhibits autophagy induction [20]. (3) Through activating ATG1 kinase, mTOR restrains autophagy induction [33]. As one of the conserved kinases, it can simultaneously promote cell growth by activating a series of metabolic pathways and by inhibiting catabolic pathways [34]. Besides autophagy, other membrane-trafficking functions are also depend on Beclin 1, so we can't affirmatively claim that autophagy suppression rather than other destruction of membrane-trafficking events contributes to tumor growth. However, *we did observe decreased cell viability and increased cell death in PCa with boosted autophagy, increased cell viability and decreased cell death with suppressed autophagy*. All of these evidences support our conclusion that autophagy is negatively correlated with tumor growth at least in some defined conditions. Together, our data suggest that (1) inhibition of PAFR selectively enhances the sensitivity of PCa cells to radiation, and (2) autophagy induction contributes to irradiation responses in PCa.

## MATERIALS AND METHODS

### Cell lines and reagents

The cell lines PC3, LNCaP, DU145 and RWPE-1 were obtained from the China Center of ATCC (Wuhan, China). Ginkgolide B (GB) was purchased from Selleck (S1343). Antibodies specific for PAFR, PARP and PCNA were obtained from Abcam (Cambridge, UK). Antibodies specific for activated caspase 3, LC3B, Beclin-1, phosphorylated Beclin 1 at Ser93 (*p*-Beclin 1), 4E-BP1, phosphorylated 4E-BP1 at Ser65 (*p*-4E-BP1), ULK1 and phosphorylated ULK1 at ser757 (*p*-ULK1) were obtained from Cell Signaling Technology (Danvers, MA, USA). PCR reagents were purchased from Roche. Other reagents were obtained from Sigma-Aldrich (St. Louis, MO, USA) except where otherwise indicated.

### Cell culture and transfection

PC3, LNCaP and DU145 were maintained in RPMI 1640 (Gibco, Shanghai) supplemented with 10% fetal bovine serum (FBS, Gibco, Melbourne) and 1% penicillin/streptomycin. RWPE-1 were maintained in Keratinocyte Serum Free Medium (K-SFM, Gibco, USA) supplemented with 1% penicillin/streptomycin. stable transfected cell line (PC3-shPAFR) was generated by transfection of psi-LVRU6MP-PAFR followed by sequential selection with 1 μmol/ml puromycin. Plasmid transfection was conducted using Lipofectamine 2000 according to the manufacturer's instructions (Invitrogen).

### Clonogenic survival assay

The experiments were conducted as described previously [15]. In brief, Cells received treatment with irradiation (or sham treatment) and PAFR inhibitor Ginkgolide B (or control vehicle). After irradiation, cells were trypsinized and resuspended in pre-warmed fresh medium containing 100 μM GB (or control treatment), counted and 5 × 10^5^ cells were plated into 2 cm^2^ culture dishes. After one to two weeks incubation, colonies with more than 50 cells were counted. The results were fitted to non-line dose-response profiles and normalized by the effects of drug treatment alone.

### Irradiation

Monolayer cells with logarithmic growth were exposed to 6 Gy (or other doses) of X-ray at ambient temperature. The control groups received sham treatment without irradiation. Radiation were delivered using a 220 kV X-ray irradiator at a dose rate of 2–3 Gy/min. After irradiation, the cells were immediately lysed for subsequent experiments or returned to thermostatic incubator for incubation.

### Western blot analysis

Immunoblotting was conducted as described previously [16]. In brief, cells were lysed in Radio Immunoprecipitation Assay (RIPA) lysis buffer and subjected to electrophoresis in 10% Bis-Tris Gel, transferred to PVDF membranes. Then, the protein expression were identified by indicated antibodies and ECL reagent (Thermo Scientific).

### RNA extraction and RT-qPCR

We extracted RNA using the RNeasy kit (Qiagen) and generated cDNA with the Transcriptor First Strand cDNA Synthesis Kit (Roche). qPCR was conducted using FastStart Universal SYBR Green Master (Roche) according to manufacturer's instructions. PAFR mRNA expression was quantified and normalized to GAPDH. PAFR (Forward: GACAGCATAGAGGCTGAGGC, Reverse: TAGCCATTAGCAATGACCCC) GAPDH (Forward: TGCACCACCAACTGCTTAGC, Reverse: GGCATGGACTGTGGTCATGAG).

### Caspase 3 activity assay

The activity of caspase 3 was measured by using the caspase 3 activity kit (Beyotime Institute of Biotechnology, China) according to the kit instructions.

### Cell cycle flow cytometry assay

Cells were fixed (70% ethanol), centrifuged (1,000g) and resuspended in PBS containing 0.05 mg/mL RNase A, and incubated at room temperature for 30 min. After incubation, the cells were rinsed three times and stained with 10 mg/ml propidium iodide followed by filtration with a 60-μm mesh. Then, 1 × 10^4^ cells were counted and analyzed by flow cytometry (FACSCalibur, BD Company) with ModFit software (Verity Software House, Inc.).

### Immunoprecipitation (CO-IP)

For CO-IP, monolayer cells were lysed and endogenous proteins were extracted by using m-Per buffer (Pierce). The lysates were incubated with PARP or Beclin 1 antibodies over night following centrifuging and the proteins were pulled down by protein A/G agarose (SantaCruz Biotechnology). After rinse, beads were dissolved in 30 ul of 2 SDS sample buffer for Western blotting.

### Tumor xenograft studies

To measure tumor formation of PC3 cells and their response to, irradiation, GB and irradiation plus GB, 6-week-old male immunocompromised NOD-SCID mice (breeding colony of Chinese Academy of Sciences) were injected subcutaneously with 10^7^ PC3 cells. Before injection, the tumor cells were embedded in Matrigel (BD Biosciences). Eight mice were injected with each group. Tumor growth was monitored by weekly measurement of tumor length (L) and width (W) and tumor volume was estimated using the formula (π/6)(LW^2^). For drug treatment, the mice were treated with either 100 mg·kg^-1^ body weight GB or isometric DMSO without GB by intraperitonially for two course of five days per week.

### Statistical analyses

One-way ANOVA was used to compare the means of two groups. Two-way ANOVA was used to compare the magnitude of changes among different conditions in more than one groups. SPSS (v.19, IBM, USA) was used to assess date. *P* < 0.05 was considered statistically significant.

## References

[1] Siegel RL, Miller KD, Jemal A (2016). Cancer statistics, 2016. CA.

[2] Zhu Y, Wang HK, Qu YY, Ye DW (2015). Prostate cancer in East Asia: evolving trend over the last decade. Asian journal of andrology.

[3] Sandler HM, Mirhadi AJ (2009). Radical radiotherapy for prostate cancer is the ‘only way to go’. Oncology.

[4] Zietman AL, Bae K, Slater JD, Shipley WU, Efstathiou JA, Coen JJ, Bush DA, Lunt M, Spiegel DY, Skowronski R, Jabola BR, Rossi CJ (2010). Randomized trial comparing conventional-dose with high-dose conformal radiation therapy in early-stage adenocarcinoma of the prostate: long-term results from proton radiation oncology group/american college of radiology 95–09. Journal of clinical oncology.

[5] Pahlajani N, Ruth KJ, Buyyounouski MK, Chen DY, Horwitz EM, Hanks GE, Price RA, Pollack A (2012). Radiotherapy doses of 80 Gy and higher are associated with lower mortality in men with Gleason score 8 to 10 prostate cancer. International journal of radiation oncology, biology, physics.

[6] Matzinger O, Duclos F, van den Bergh A, Carrie C, Villa S, Kitsios P, Poortmans P, Sundar S, van der Steen-Banasik EM, Gulyban A, Collette L, Bolla M, Group ERO (2009). Acute toxicity of curative radiotherapy for intermediate- and high-risk localised prostate cancer in the EORTC trial 22991. European journal of cancer.

[7] Pollack A, Zagars GK, Starkschall G, Antolak JA, Lee JJ, Huang E, von Eschenbach AC, Kuban DA, Rosen I (2002). Prostate cancer radiation dose response: results of the M. D. Anderson phase III randomized trial. International journal of radiation oncology, biology, physics.

[8] Valerie NC, Casarez EV, Dasilva JO, Dunlap-Brown ME, Parsons SJ, Amorino GP, Dziegielewski J. (2011). Inhibition of neurotensin receptor 1 selectively sensitizes prostate cancer to ionizing radiation. Cancer research.

[9] Nikitenko LL, Leek R, Henderson S, Pillay N, Turley H, Generali D, Gunningham S, Morrin HR, Pellagatti A, Rees MC, Harris AL, Fox SB. (2013). The G-protein-coupled receptor CLR is upregulated in an autocrine loop with adrenomedullin in clear cell renal cell carcinoma and associated with poor prognosis. Clinical cancer research.

[10] Chen J, Lan T, Zhang W, Dong L, Kang N, Zhang S, Fu M, Liu B, Liu K, Zhan Q (2015). Feed-Forward Reciprocal Activation of PAFR and STAT3 Regulates Epithelial-Mesenchymal Transition in Non-Small Cell Lung Cancer. Cancer research.

[11] Chen J, Lan T, Zhang W, Dong L, Kang N, Zhang S, Fu M, Liu B, Liu K, Zhang C, Hou J, Zhan Q (2015). Platelet-activating factor receptor-mediated PI3K/AKT activation contributes to the malignant development of esophageal squamous cell carcinoma. Oncogene.

[12] Yu Y, Zhang M, Zhang X, Cai Q, Zhu Z, Jiang W, Xu C. (2014). Transactivation of epidermal growth factor receptor through platelet-activating factor/receptor in ovarian cancer cells. Journal of experimental & clinical cancer research.

[13] Yu Y, Zhang X, Hong S, Zhang M, Cai Q, Zhang M, Jiang W, Xu C (2014). The expression of platelet-activating factor receptor modulates the cisplatin sensitivity of ovarian cancer cells: a novel target for combination therapy. British journal of cancer.

[14] Xu B, Gao L, Wang L, Tang G, He M, Yu Y, Ni X, Sun Y. (2013). Effects of platelet-activating factor and its differential regulation by androgens and steroid hormones in prostate cancers. British journal of cancer.

[15] Dziegielewski J, Baulch JE, Goetz W, Coleman MC, Spitz DR, Murley JS, Grdina DJ, Morgan WF. (2008). WR-1065, the active metabolite of amifostine, mitigates radiation-induced delayed genomic instability. Free radical biology & medicine.

[16] Yao B, Zhao J, Li Y, Li H, Hu Z, Pan P, Zhang Y, Du E, Liu R, Xu Y (2015). Elf5 inhibits TGF-beta-driven epithelial-mesenchymal transition in prostate cancer by repressing SMAD3 activation. The Prostate.

[17] Nazio F, Strappazzon F, Antonioli M, Bielli P, Cianfanelli V, Bordi M, Gretzmeier C, Dengjel J, Piacentini M, Fimia GM, Cecconi F. (2013). mTOR inhibits autophagy by controlling ULK1 ubiquitylation, self-association and function through AMBRA1 and TRAF6. Nature cell biology.

[18] Yu L, McPhee CK, Zheng L, Mardones GA, Rong Y, Peng J, Mi N, Zhao Y, Liu Z, Wan F, Hailey DW, Oorschot V, Klumperman J (2010). Termination of autophagy and reformation of lysosomes regulated by mTOR. Nature.

[19] Kim YC, Guan KL (2015). mTOR: a pharmacologic target for autophagy regulation. The Journal of clinical investigation.

[20] Wei Y, Zou Z, Becker N, Anderson M, Sumpter R, Xiao G, Kinch L, Koduru P, Christudass CS, Veltri RW, Grishin NV, Peyton M, Minna J (2013). EGFR-mediated Beclin 1 phosphorylation in autophagy suppression, tumor progression, and tumor chemoresistance. Cell.

[21] Denizot Y, Descottes B, Truffinet V, Valleix D, Labrousse F, Mathonnet M (2005). Platelet-activating factor and liver metastasis of colorectal cancer. International journal of cancer.

[22] Im SY, Ko HM, Kim JW, Lee HK, Ha TY, Lee HB, Oh SJ, Bai S, Chung KC, Lee YB, Kang HS, Chun SB. (1996). Augmentation of tumor metastasis by platelet-activating factor. Cancer research.

[23] Kang YH, Kim WH, Park MK, Han BH (2001). Antimetastatic and antitumor effects of benzoquinonoid AC7–1 from Ardisia crispa. International journal of cancer Journal international du cancer.

[24] Zhao Y, Wang Q, Qiu G, Zhou S, Jing Z, Wang J, Wang W, Cao J, Han K, Cheng Q, Shen B, Chen Y, Zhang WJ (2015). RACK1 Promotes Autophagy by Enhancing the Atg14L-Beclin 1-Vps34-Vps15 Complex Formation upon Phosphorylation by AMPK. Cell reports.

[25] Wauson EM, Zaganjor E, Lee AY, Guerra ML, Ghosh AB, Bookout AL, Chambers CP, Jivan A, McGlynn K, Hutchison MR, Deberardinis RJ, Cobb MH (2012). The G protein-coupled taste receptor T1R1/T1R3 regulates mTORC1 and autophagy. Molecular cell.

[26] Wauson EM, Zaganjor E, Cobb MH (2013). Amino acid regulation of autophagy through the GPCR TAS1R1-TAS1R3. Autophagy.

[27] Cao C, Subhawong T, Albert JM, Kim KW, Geng L, Sekhar KR, Gi YJ, Lu B (2006). Inhibition of mammalian target of rapamycin or apoptotic pathway induces autophagy and radiosensitizes PTEN null prostate cancer cells. Cancer research.

[28] Zhuang W, Li B, Long L, Chen L, Huang Q, Liang Z (2011). Induction of autophagy promotes differentiation of glioma-initiating cells and their radiosensitivity. International journal of cancer.

[29] Lomonaco SL, Finniss S, Xiang C, Decarvalho A, Umansky F, Kalkanis SN, Mikkelsen T, Brodie C. (2009). The induction of autophagy by gamma-radiation contributes to the radioresistance of glioma stem cells. International journal of cancer.

[30] Wang RC, Wei Y, An Z, Zou Z, Xiao G, Bhagat G, White M, Reichelt J, Levine B (2012). Akt-mediated regulation of autophagy and tumorigenesis through Beclin 1 phosphorylation. Science.

[31] Levine B, Kroemer G (2008). Autophagy in the pathogenesis of disease. Cell.

[32] Hanahan D, Weinberg RA. (2011). Hallmarks of cancer: the next generation. Cell.

[33] Diaz-Troya S, Perez-Perez ME, Florencio FJ, Crespo JL (2008). The role of TOR in autophagy regulation from yeast to plants and mammals. Autophagy.

[34] Laplante M, Sabatini DM (2012). mTOR signaling in growth control and disease. Cell.

